# High-Resolution Melting Curve Analysis for Identification of *Pasteurellaceae* Species in Experimental Animal Facilities

**DOI:** 10.1371/journal.pone.0142560

**Published:** 2015-11-10

**Authors:** Manuel Miller, Julia Zorn, Markus Brielmeier

**Affiliations:** Research Unit Comparative Medicine, German Research Center for Environmental Health, Neuherberg, Germany; Chang Gung University, TAIWAN

## Abstract

*Pasteurellaceae* are among the most prevalent bacterial pathogens isolated from mice housed in experimental animal facilities. Reliable detection and differentiation of *Pasteurellaceae* are essential for high-quality health monitoring. In this study, we combined a real-time PCR assay amplifying a variable region in the 16S rRNA sequence with high-resolution melting curve analysis (HRM) to identify and differentiate among the commonly isolated species *Pasteurella pneumotropica* biotypes “Jawetz” and “Heyl”, *Actinobacillus muris*, and *Haemophilus influenzaemurium*. We used a set of six reference strains for assay development, with the melting profiles of these strains clearly distinguishable due to DNA sequence variations in the amplicon. For evaluation, we used real-time PCR/HRM to test 25 unknown *Pasteurellaceae* isolates obtained from an external diagnostic laboratory and found the results to be consistent with those of partial 16S rRNA sequencing. The real-time PCR/HRM method provides a sensitive, rapid, and closed-tube approach for *Pasteurellaceae* species identification for health monitoring of laboratory mice.

## Introduction

The *Pasteurellaceae* family consists of several genera, of which *Pasteurella pneumotropica* biotypes “Jawetz” and “Heyl”, *Actinobacillus muris*, and *Haemophilus influenzaemurium* are regularly identified during routine health monitoring of laboratory mice. *Pasteurellaceae* constitute the most prevalent bacterial pathogens in experimental facilities worldwide [1,2]. Although the pathogenicity of most *Pasteurellaceae* species is low, *P*. *pneumotropica* is associated with variable clinical manifestations such as infections of the eye, genital tract, and respiratory system [3]. Even subclinical *P*. *pneumotropica* infections in immunocompetent mice could represent an unwanted experimental variable and may influence results [4]. Recently, the Federation of European Laboratory Animal Science Associations revised their recommendations on *Pasteurellaceae* reporting. While the prior version recommended monitoring all *Pasteurellaceae* species, specific identification of *P*. *pneumotropica* is currently recommended [5]. However, depending upon the research focus, some facilities may include identification of other *Pasteurellaceae* species, although this is controversial. Identification of *Pasteurellaceae* during routine health monitoring should be reliable, sensitive, fast, and easy to perform. However, no currently available method fulfills these criteria. *Pasteurellaceae* are usually detected by swab samples from the nasopharynx, genital tract, or large intestine and are cultured on agar plates, with subsequent analysis of suspicious colonies by using biochemical test kits. However, commonly used kits are optimized for human samples, and are not only unreliable for rodent sample identification to the species level but also sometimes fail to identify the correct bacterial family [5–7]. Analysis of subcultured bacterial colonies by matrix-assisted laser desorption/ionization-time of flight mass spectrometry offers good specificity; however, this method is expensive and lacks appropriate murine datasets. Additionally, serological tests are inappropriate for diagnosis of *Pasteurellaceae* infections, given that seroconversion of sentinel mice exposed to contaminated bedding or cohabitating with infected mice is unreliable [8]. There are also PCR-based options available for detection and differentiation of rodent-specific *Pasteurellaceae*: a genus-specific PCR assay to detect all *Pasteurellaceae* [9], species-specific assays to detect either of the *P*. *pneumotropica* biotypes “Jawetz” or “Heyl” [10,11], a multiplex PCR assay based on the 16S-23S rRNA internal-transcribed spacer region [12], and a real-time PCR assay that distinguishes between the *P*. *pneumotropica* biotypes “Heyl” and “Jawetz” [13]. Unfortunately, only a limited number of species can be identified by multiplex PCR assays, and the specific real-time PCR assay for *P*. *pneumotropica* “Jawetz” has a low discrimination potential against *A*. *muris*. PCR followed by fragment sequencing provides an accurate and reliable identification method. However, it is time consuming and expensive. The combination of real-time PCR assays with high-resolution melting curve analysis (HRM) [14] offers a relatively new molecular technique for genotyping, mutation scanning, sequence matching [15], and identification of bacterial species [16]. HRM analysis of the 16S rRNA gene, as well as other highly conserved genes, successfully identified microbial isolates, including *Mycoplasma* [17], *Listeria* [18–19], *Brucella* [20], *Lactobacillus* [21], and *Staphylococcus* [22] to the species or strain level. Recently, the first multiplex HRM real-time PCR assay was developed for simultaneous detection of *Salmonella*, *Listeria monocytogenes*, and *Staphylococcus aureus* [23]. The aim of this study was to develop a reliable, simple, and rapid alternative method to identify *P*. *pneumotropica* and other common *Pasteurellaceae* species by using a real-time PCR assay with HRM analysis.

## Materials and Methods

### Bacterial strains and isolates

Six *Pasteurellaceae* reference strains were used for assay development and as positive controls in each experiment (Table 1). Four were obtained from different culture collections (German Collection of Microorganisms and Cell Cultures: NCTC 8141 and NCTC 12432; Collection of the Pasteur Institute: CNP 160; Public Health England: NCTC 11146). The remaining two isolates included strains isolated and characterized within our facility (HMGU isolates). To evaluate the reference strains against a library of unknown isolates, 25 *Pasteurellaceae* isolates were used. These isolates were obtained from an external diagnostic laboratory and had been isolated from nasopharyngeal, tracheal, genital, or intestinal swab specimens from mice during routine health monitoring procedures.

**Table 1:** Overview of reference species and assignment of clinical isolates.

				T_m_ (°C)^a^	
Reference species (*n* = 6)	Reference strain	GenBank accession number	No. of isolates (*n* = 25)^b^	Mean	SD
*Pasteurella pneumotropica* "Jawetz"	NCTC 8141	M75083	9	76.78	0.16
*Pasteurella pneumotropica* "Heyl"	CNP 160	AF012090	4	79.10	0.13
*Pasteurella pneumotropica*	HMGU isolate	HMGU isolate	1	78.77	0.14
*Actinobacillus muris*	NCTC 12432	NR_042870	3	77.82	0.15
*Actinobacillus muris*	HMGU isolate	HMGU isolate	6	78.35	0.13
*Haemophilus influenzaemurium*	NCTC 11146	AF024530	2	78.56	0.17

^a^ Mean T_m_ and standard deviation (SD) were generated using T_m_ values from reference strains analyzed in quadruplicate by four real-time PCR/HRM experiments over a 1-year period.

^b^ Number of isolates assigned to the respective reference strain by real-time PCR/HRM.

### Bacterial culture

Isolates from the external sources were delivered in brain heart infusion broth (bioMérieux, Nürtingen, Germany) and subcloned for 24 h at 37°C on Columbia agar with 5% sheep blood (VWR International, Darmstadt, Germany). Single colonies were picked and propagated in brain heart infusion broth (Sigma-Aldrich, Munich, Germany) for 24 h at 37°C for DNA extraction. Liquid culture (1 mL) was processed using the DNeasy Blood and Tissue Kit (Qiagen, Hilden, Germany), according to manufacturer instructions.

### Sequencing

Genotypic analysis of all isolates was undertaken by partial 16S rRNA gene sequencing following genus-specific PCR using the primers 5′-CATAAGATGAGCCCAAG-3′ (forward) and 5′-GTCAGTACATTCCCAAGG-3′ (reverse) surrounding a 530-bp fragment [9]. PCR products were purified using the MinElute PCR Purification Kit (Qiagen) and sequenced at GATC Biotech AG (Konstanz, Germany). Sequence assignment was performed following a homology search using the Basic Local Alignment Search Tool provided by the National Centre for Biotechnology Information (http://www.ncbi.nlm.nih.gov).

### Real-time PCR and HRM

A real-time PCR assay [13] using the forward primer 5′-CGGGTTGTAAAGTTCTTTCGGT-3′ and reverse primer 5′-GGAGTTAGCCGGTGCTTCTTC-3′ was optimized for the RotorGene Q instrument (Qiagen). The primers flank a highly variable region of the 16S rRNA gene from the *Pasteurellaceae* family, with the resulting 93-bp amplicons used to generate the discriminating melting curves. Bacterial genomic DNA templates (4 ng) were added to a reaction mixture consisting of 10 μL 2X Type-it HRM Master Mix (Qiagen) containing EvaGreen DNA-binding dye, 720 nM of each primer, and ultrapure water to a final reaction volume of 20 μL. The PCR thermocycling parameters were as follows: initial denaturation at 95°C for 5 min, 40 cycles with denaturation at 95°C for 10 s and annealing/extension at 58°C for 30 s, followed by HRM ramping from 73–83°C. Fluorescence data were acquired at 0.1°C increments every 2 s in order to generate specific melting curves. For each experiment, the six reference strains were included as melting curve standards. Reactions were performed in quadruplicate for reference strains and triplicate for unknown isolates. The resulting melting curves were normalized to relative values of 100% (pre-melting phase) or 0% (post-melting phase) to eliminate differences in fluorescence intensity and background fluorescence between wells. To exclude contaminations in the reaction mixture, a no-template control was added to each experiment. Data analysis was performed using the Rotor-Gene Q Software 2.1 (Qiagen)

## Results

### HRM of reference strains

**Fig 1:**
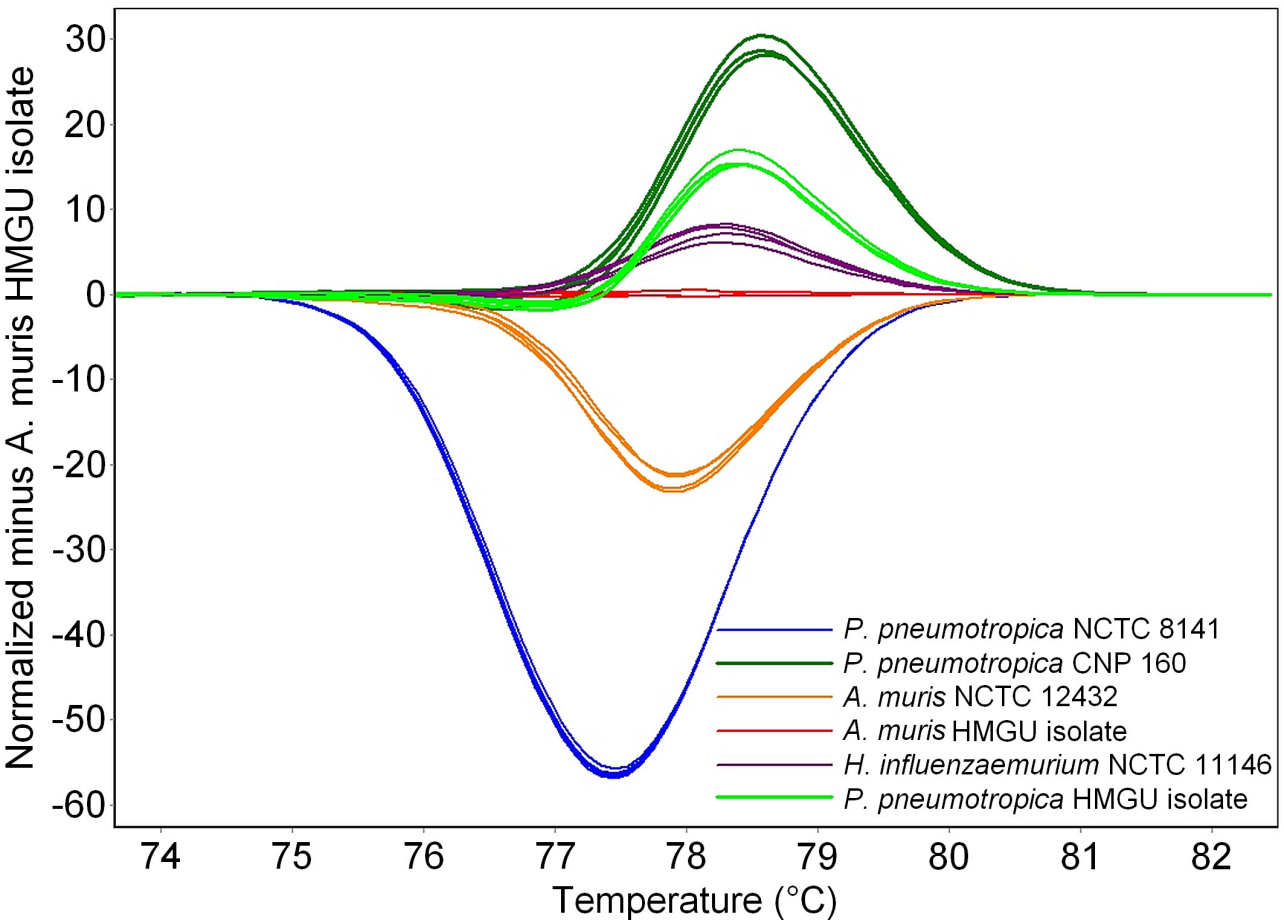
High-resolution melting analysis of six *Pasteurellaceae* reference strains. Representative difference curves derived from the normalized data using the *Actinobacillus muris* (HMGU isolate) reference as the baseline.

We assessed the suitability of the assay to distinguish between different *Pasteurellaceae* species and strains by testing the reference strains obtained from different culture collections or isolated from mice within our facility. Each reference strain exhibited a distinct HRM profile (Fig 1). The amplicons subjected to high-resolution melt generated melting peaks (T_m_) ranging from 76.78°C to 79.10°C. The melting profiles of all strains resulted in a single peak with a maximum T_m_ variation of 0.1°C for each of the quadruplicates. Standard deviations within individual experiments ranged from 0.01 to 0.06 for all reference strains. Since the mean T_m_ values for the six strains differed by at least 0.21°C, they were deemed suitable for discriminating between the reference strains. Reproducibility was confirmed by analyzing the reference strains in four experiments (Table 1). The GC content of the six amplicons ranged from 43% to 47%, with amplicons having the same GC content displaying different melting points based on sequence differences. The *P*. *pneumotropica* “Heyl” and “HMGU isolate” reference strains differed in only one base pair (C/G–T/A). This single-base modification induced a 0.33°C shift in melting point.

### Analysis of unknown isolates by HRM and sequencing

To validate the assay in a relevant setting, 25 unknown *Pasteurellaceae* isolates were obtained from an external laboratory animal diagnostics provider. The generated melting points for each of the 25 isolates matched one of the reference strains. All 6 reference melting points were observed among the 25 isolates (Table 1). HRM identification results were compared to partial 16S rRNA gene-sequencing results. Fragments (at least 300 bp) spanning the real-time PCR/HRM amplicon were used to categorize the isolates with one of the reference strains on the basis of similarity in their respective gene sequences, as obtained from GenBank. All isolates showed a minimum 97% shared sequence identity with one of the reference sequences. Twenty-three isolates displayed 100% similarity to their reference strain when the 93-bp real-time PCR/HRM amplicons were used for alignment. Two *H*. *influenzaemurium* isolates displayed 98% sequence similarity to the related reference strain based on two opposite and adjacent single-nucleotide modifications (A/G and G/A vs. G/A and A/G), which did not result in a change in the melting temperature. The multiple sequence alignment of the real-time PCR/HRM amplicons of reference strains and clinical isolates is shown in S1 Dataset.

## Discussion

In this study, we described a new approach using a real-time PCR/HRM system for identification and discrimination of *Pasteurellaceae* species, one of the most prevalent bacterial pathogens found in laboratory mice. The six reference species used for assay development exhibited sufficiently distinguishable melting profiles based on their different T_m_ values. Evaluation of the newly developed method using 25 unknown *Pasteurellaceae* isolates revealed accurate species identification of all specimens, confirmed by partial 16S rRNA sequencing. While sequencing is expensive and time consuming, the present method allows PCR and sequence analysis in a single step and can be completed in a few hours, thereby considerably increasing testing speed. Another advantage of this closed-tube method is reduced sample handling, resulting in elimination of PCR product contamination. Here, we used a short amplicon, because single-nucleotide modifications indicate unique melting points, even when specimens have the same GC content. Larger amplicons containing regions with different GC content may allow species differentiation based on changes in the melting curve profile. However, the effectiveness of detecting single-base substitutions decreases with increasing DNA-sequence length. In this study, sequence analysis revealed two opposite and adjacent single-nucleotide modifications in both *H*. *influenzaemurium* isolates as compared to the reference strain sequence. Species-level identification by HRM was reliable; however, this case revealed a major limitation of HRM. An unknown specimen not included in the reference library may possess the same T_m_ value as a non-associated reference strain. In this case, the melting point would allow assignment of the wrong species. This situation could only be avoided by rRNA sequencing, although accurate primer design might minimize the risk. Unknown strains with unprecedented melting profiles could be identified by sequencing and then be incorporated as additional reference controls. Here, only the application of pure isolated DNA from a single isolate yielded precise results. Based on these results, the combination of real-time PCR and HRM represents a fast and efficient application for the identification of *Pasteurellaceae* isolates grown from swab samples collected during health monitoring procedures. This simple and low-cost method has great potential to become a useful tool for research and diagnostic laboratories and can be easily adapted to other pathogenic bacteria.

## Supporting Information

S1 Dataset Multiple sequence alignment of the real-time PCR/HRM amplicons of reference strains and clinical isolates. (PDF)

## References

[1] Pritchett-Corning KR, Cosentino J, Clifford CB. Contemporary prevalence of infectious agents in laboratory mice and rats. Lab Anim. 2009; 43: 165–173. 10.1258/la.2008.008009 19015179

[2] Hayashimoto N, Morita H, Ishida T, Yasuda M, Kameda S, Uchida R, et al Current microbiological status of laboratory mice and rats in experimental facilities in Japan. Exp Anim. 2013; 62: 41–48. 23357945 10.1538/expanim.62.41

[3] Council NR. Infectious diseases of mice and rats. Washington DC: The National Academies Press; 1991. 415 p. 25144101

[4] Patten CC Jr., Myles MH, Franklin CL, Livingston RS. Perturbations in cytokine gene expression after inoculation of C57BL/6 mice with *Pasteurella pneumotropica* . Comp Med. 2010; 60: 18–24. 20158944 PMC2826080

[5] Mähler Convenor M, Berard M, Feinstein R, Gallagher A, Illgen-Wilcke B, Pritchett-Corning K, et al FELASA recommendations for the health monitoring of mouse, rat, hamster, guinea pig and rabbit colonies in breeding and experimental units. Lab Anim. 2014; 48: 178–192. 10.1177/0023677213516312 24496575

[6] Hayashimoto N, Aiba T, Itoh K, Kato M, Kawamoto E, Kiyokawa S, et al Identification procedure for *Pasteurella pneumotropica* in microbiologic monitoring of laboratory animals. Exp Anim. 2005; 54: 123–129. 15897620 10.1538/expanim.54.123

[7] Boot R, Van den Brink M, Handgraaf P, Timmermans R. The use of the API 20 NE bacteria classification procedure to identify *Pasteurellaceae* strains in rodents and rabbits. Scand J Lab Anim Sci. 2004; 31: 177–183.

[8] Scharmann W, Heller A. Survival and transmissibility of *Pasteurella pneumotropica* . Lab Anim. 2001; 35: 163–166. 11315166 10.1258/0023677011911543

[9] Bootz F, Kirschnek S, Nicklas W, Wyss SK, Homberger FR. Detection of *Pasteurellaceae* in rodents by polymerase chain reaction analysis. Lab Anim Sci. 1998; 48: 542–546. 10090074

[10] Wang RF, Campbell W, Cao WW, Summage C, Steele RS, Cerniglia CE. Detection of *Pasteurella pneumotropica* in laboratory mice and rats by polymerase chain reaction. Lab Anim Sci. 1996; 46: 81–85. 8699827

[11] Kodjo A, Villard L, Veillet F, Escande F, Borges E, Maurin F, et al Identification by 16S rDNA fragment amplification and determination of genetic diversity by random amplified polymorphic DNA analysis of *Pasteurella pneumotropica* isolated from laboratory rodents. Lab Anim Sci. 1999; 49: 49–53. 10090094

[12] Benga L, Benten WP, Engelhardt E, Bleich A, Gougoula C, Sager M. Development of a multiplex PCR assay based on the 16S-23S rRNA internal transcribed spacer for the detection and identification of rodent *Pasteurellaceae* . J Microbiol Methods. 2013; 95: 256–261. 10.1016/j.mimet.2013.09.005 24055385

[13] Dole VS, Banu LA, Fister RD, Nicklas W, Henderson KS. Assessment of rpoB and 16S rRNA genes as targets for PCR-based identification of *Pasteurella pneumotropica* . Comp Med. 2010; 60: 427–435. 21262128 PMC3002101

[14] Wittwer CT, Reed GH, Gundry CN, Vandersteen JG, Pryor RJ. High-resolution genotyping by amplicon melting analysis using LCGreen. Clin Chem. 2003; 49: 853–860. 12765979 10.1373/49.6.853

[15] Reed GH, Kent JO, Wittwer CT. High-resolution DNA melting analysis for simple and efficient molecular diagnostics. Pharmacogenomics. 2007; 8: 597–608. 10.2217/14622416.8.6.597 17559349

[16] Cheng JC, Huang CL, Lin CC, Chen CC, Chang YC, Chang SS, et al Rapid detection and identification of clinically important bacteria by high-resolution melting analysis after broad-range ribosomal RNA real-time PCR. Clin Chem. 2006; 52: 1997–2004. 10.1373/clinchem.2006.069286 16990426

[17] Rebelo AR, Parker L, Cai HY. Use of high-resolution melting curve analysis to identify *Mycoplasma* species commonly isolated from ruminant, avian, and canine samples. J Vet Diagn Invest. 2011; 23: 932–936 10.1177/1040638711416846 21908349

[18] Ohshima C, Takahashi H, Phraephaisarn C, Vesaratchavest M, Keeratipibul S, Kuda T, et al Establishment of a simple and rapid identification method for *Listeria* spp. by using high-resolution melting analysis, and its application in food industry. PLoS One. 2014; 9: e99223 10.1371/journal.pone.0099223 24918440 PMC4053416

[19] Sakaridis I, Ganopoulos I, Madesis P, Tsaftaris A, Argiriou A. Genotyping of *Listeria monocytogenes* isolates from poultry carcasses using high resolution melting (HRM) analysis. Biotechnol Biotechnol Equip. 2014; 28: 107–111. 10.1080/13102818.2014.901681 26019495 PMC4433902

[20] Mohamed Zahidi Ja, Bee Yong T, Hashim R, Mohd Noor A, Hamzah SH, Ahmad N. Identification of *Brucella* spp. isolated from human brucellosis in Malaysia using high-resolution melt (HRM) analysis. Diagn Microbiol Infect Dis. 2015; 81: 227–233. 10.1016/j.diagmicrobio.2014.12.012 25641125

[21] Iacumin L, Ginaldi F, Manzano M, Anastasi V, Reale A, Zotta T, et al High resolution melting analysis (HRM) as a new tool for the identification of species belonging to the *Lactobacillus casei* group and comparison with species-specific PCRs and multiplex PCR. Food Microbiol. 2015; 46: 357–367. 10.1016/j.fm.2014.08.007 25475306

[22] Chen JH, Cheng VC, Chan JF, She KK, Yan MK, Yau MC, et al The use of high-resolution melting analysis for rapid spa typing on methicillin-resistant *Staphylococcus aureus* clinical isolates. J Microbiol Methods. 2013; 92: 99–102. 10.1016/j.mimet.2012.11.006 23154043

[23] Xiao X-l, Zhang L, Wu H, Yu Y-g, Tang Y-q, Liu D-m, et al Simultaneous detection of *Salmonella*, *Listeria monocytogenes*, and *Staphylococcus aureus* by multiplex real-time PCR assays using high-resolution melting. Food Anal Methods. 2014; 7: 1960–1972. 10.1007/s12161-014-9875-x

