# Supplementary material for: High-Resolution Melting Curve Analysis for Identification of Pasteurellaceae Species in Experimental Animal Facilities
Source: PLoS One. 2015 Nov 10;10(11):e0142560. doi: 10.1371/journal.pone.0142560 (PMC4640598; doi:10.1371/journal.pone.0142560)
Supplement: S1 Dataset — (PDF) [file pone.0142560.s001.pdf]

Supporting information

S1 Dataset. Multiple sequence alignment of the real-time PCR/HRM amplicons of reference strains and clinical isolates.

```
References
P.p. NCTC 8141    CGGGTTGTAAAGTTCTTTCGGTGATGAGGAAGGTGATAAGGTTAATACCCTTATTAATTGACGTTAGTCACAGAAGAAGCACCGGCTAACTCC
P.p. CP160        CGGGTTGTAAAGTTCTTTCGGTGATGAGGAAGGCAGTTTGGTTAATAGCCAAGCTGATTGACGTTAGTCACAGAAGAAGCACCGGCTAACTCC
A.m. NCTC 12432   CGGGTTGTAAAGTTCTTTCGGTGATGAGGAAGGTGTAATGGTTAATACCCGTTACAATTGACGTTAGTCACAGAAGAAGCACCGGCTAACTCC
A.m. HMGU isolate CGGGTTGTAAAGTTCTTTCGGTGATGAGGAAGGTGTGATGGTTAATACCCATGACAATTGACGTTAGTCACAGAAGAAGCACCGGCTAACTCC
H.i. NCTC 11146   CGGGTTGTAAAGTTCTTTCGGTGATGAGGAAGGCAGCTTNGTTAATACCCAAGTTNATTGACGTTAATCACAGAAGAAGCACCGGCTAACTCC
P.p. HMGU isolate CGGGTTGTAAAGTTCTTTCGGTGATGAGGAAGGCAGTTTGGTTAATAGCCAAGTTGATTGACGTTAGTCACAGAAGAAGCACCGGCTAACTCC

Isolates
No.1              CGGGTTGTAAAGTTCTTTCGGTGATGAGGAAGGTGATAAGGTTAATACCCTTATTAATTGACGTTAGTCACAGAAGAAGCACCGGCTAACTCC
No.2              CGGGTTGTAAAGTTCTTTCGGTGATGAGGAAGGTGTAATGGTTAATACCCGTTACAATTGACGTTAGTCACAGAAGAAGCACCGGCTAACTCC
No.3              CGGGTTGTAAAGTTCTTTCGGTGATGAGGAAGGCAGTTTGGTTAATAGCCAAGCTGATTGACGTTAGTCACAGAAGAAGCACCGGCTAACTCC
No.4              CGGGTTGTAAAGTTCTTTCGGTGATGAGGAAGGTGATAAGGTTAATACCCTTATTAATTGACGTTAGTCACAGAAGAAGCACCGGCTAACTCC
No.5              CGGGTTGTAAAGTTCTTTCGGTGATGAGGAAGGTGATAAGGTTAATACCCTTATTAATTGACGTTAGTCACAGAAGAAGCACCGGCTAACTCC
No.6              CGGGTTGTAAAGTTCTTTCGGTGATGAGGAAGGCGACTTGGTTAATACCCAAGTTGATTGACGTTAATCACAGAAGAAGCACCGGCTAACTCC
No.7              CGGGTTGTAAAGTTCTTTCGGTGATGAGGAAGGCGACTTGGTTAATACCCAAGTTGATTGACGTTAATCACAGAAGAAGCACCGGCTAACTCC
No.8              CGGGTTGTAAAGTTCTTTCGGTGATGAGGAAGGTGATAAGGTTAATACCCTTATTAATTGACGTTAGTCACAGAAGAAGCACCGGCTAACTCC
No.9              CGGGTTGTAAAGTTCTTTCGGTGATGAGGAAGGTGATAAGGTTAATACCCTTATTAATTGACGTTAGTCACAGAAGAAGCACCGGCTAACTCC
No.10             CGGGTTGTAAAGTTCTTTCGGTGATGAGGAAGGTGATAAGGTTAATACCCTTATTAATTGACGTTAGTCACAGAAGAAGCACCGGCTAACTCC
No.11             CGGGTTGTAAAGTTCTTTCGGTGATGAGGAAGGCAGTTTGGTTAATAGCCAAGTTGATTGACGTTAGTCACAGAAGAAGCACCGGCTAACTCC
No.12             CGGGTTGTAAAGTTCTTTCGGTGATGAGGAAGGCAGTTTGGTTAATAGCCAAGCTGATTGACGTTAGTCACAGAAGAAGCACCGGCTAACTCC
No.13             CGGGTTGTAAAGTTCTTTCGGTGATGAGGAAGGTGTAATGGTTAATACCCGTTACAATTGACGTTAGTCACAGAAGAAGCACCGGCTAACTCC
No.14             CGGGTTGTAAAGTTCTTTCGGTGATGAGGAAGGTGATAAGGTTAATACCCTTATTAATTGACGTTAGTCACAGAAGAAGCACCGGCTAACTCC
No.15             CGGGTTGTAAAGTTCTTTCGGTGATGAGGAAGGTGTGATGGTTAATACCCATGACAATTGACGTTAGTCACAGAAGAAGCACCGGCTAACTCC
No.16             CGGGTTGTAAAGTTCTTTCGGTGATGAGGAAGGTGTGATGGTTAATACCCATGACAATTGACGTTAGTCACAGAAGAAGCACCGGCTAACTCC
No.17             CGGGTTGTAAAGTTCTTTCGGTGATGAGGAAGGCAGTTTGGTTAATAGCCAAGCTGATTGACGTTAGTCACAGAAGAAGCACCGGCTAACTCC
No.18             CGGGTTGTAAAGTTCTTTCGGTGATGAGGAAGGTGTGATGGTTAATACCCATGACAATTGACGTTAGTCACAGAAGAAGCACCGGCTAACTCC
No.19             CGGGTTGTAAAGTTCTTTCGGTGATGAGGAAGGTGATAAGGTTAATACCCTTATTAATTGACGTTAGTCACAGAAGAAGCACCGGCTAACTCC
No.20             CGGGTTGTAAAGTTCTTTCGGTGATGAGGAAGGTGTGATGGTTAATACCCATGACAATTGACGTTAGTCACAGAAGAAGCACCGGCTAACTCC
No.21             CGGGTTGTAAAGTTCTTTCGGTGATGAGGAAGGTGTAATGGTTAATACCCGTTACAATTGACGTTAGTCACAGAAGAAGCACCGGCTAACTCC
No.22             CGGGTTGTAAAGTTCTTTCGGTGATGAGGAAGGTGATAAGGTTAATACCCTTATTAATTGACGTTAGTCACAGAAGAAGCACCGGCTAACTCC
No.23             CGGGTTGTAAAGTTCTTTCGGTGATGAGGAAGGTGTGATGGTTAATACCCATGACAATTGACGTTAGTCACAGAAGAAGCACCGGCTAACTCC
No.24             CGGGTTGTAAAGTTCTTTCGGTGATGAGGAAGGCAGTTTGGTTAATAGCCAAGCTGATTGACGTTAGTCACAGAAGAAGCACCGGCTAACTCC
No.25             CGGGTTGTAAAGTTCTTTCGGTGATGAGGAAGGTGTGATGGTTAATACCCATGACAATTGACGTTAGTCACAGAAGAAGCACCGGCTAACTCC
                  *********************************vvvvvv********v**vvvvvv**********v**************************
```

* = identical base in all aligned sequences; v = variable base that leads to a T_m shift
